# Identification of Genes Affecting the Toxicity of Anti-Cancer Drug Bortezomib by Genome-Wide Screening in *S. pombe*

**DOI:** 10.1371/journal.pone.0022021

**Published:** 2011-07-08

**Authors:** Kojiro Takeda, Ayaka Mori, Mitsuhiro Yanagida

**Affiliations:** G0 Cell Unit, Okinawa Institute of Science and Technology (OIST), Okinawa, Japan; Texas A&M University, United States of America

## Abstract

Bortezomib/PS-341/Velcade, a proteasome inhibitor, is widely used to treat multiple myeloma. While several mechanisms of the cytotoxicity of the drug were proposed, the actual mechanism remains elusive. We aimed to identify genes affecting the cytotoxicity of Bortezomib in the fission yeast *S.pombe* as the drug inhibits this organism's cell division cycle like proteasome mutants. Among the 2815 genes screened (covering 56% of total ORFs), 19 genes, whose deletions induce strong synthetic lethality with Bortezomib, were identified. The products of the 19 genes included four ubiquitin enzymes and one nuclear proteasome factor, and 13 of them are conserved in humans. Our results will provide useful information for understanding the actions of Bortezomib within cells.

## Introduction

The ubiquitin/proteasome pathway is a major proteolytic machinery in cells and has pivotal roles in the cell division cycle, apoptosis, etc [1]. Therefore, this pathway is considered to be a strong potential target of clinical treatment for diseases such as cancer, and chemicals that modulate the activity of the ubiquitin/proteasome pathway have been intensively investigated [2], [3]. Bortezomib/PS-341/Velcade is a peptide boronic acid that inhibits the chymotrypsin-like activity of the beta 5 subunit of the proteasome *in vitro*. Bortezomib has strong potential anti-tumor effects in *in vitro* and animal studies and has been developed as an anti-cancer drug to treat multiple myeloma and other cancers [3], [4]. Inhibiting the proteasome causes pleiotropic effects. Therefore, several mechanisms for the cytotoxicity of Bortezomib have been suggested, like inhibition of anti-apoptotic proteins, stabilization of p53, disturbance of cell cycle progression, etc [5]. To increase knowledge of the mechanisms of the anti-tumor and adverse effects of Bortezomib, it will be beneficial to identify genes involving in the cytotoxicity of this drug. A pioneer effort to identify such genes was reported by Lightcap group in 2010 [6].

The fission yeast *Schizosaccharomyces pombe* is a simple unicellular eukaryote and has been used as a model organism of basic cell biology, owing to its genetic tractability and its similarity to higher eukaryotes. A library of 2815 gene-deleted strains is available for genome-wide studies of drug sensitivity [7], [8], [9].

Here, we attempted to identify evolutionally conserved genes affecting the cytotoxicity of Bortezomib by taking advantage of the gene-deletion library in *S. pombe* and established a method to perform genome-wide synthetic lethal screening with Bortezomib. Among the 2815 genes screened, deletion strains of 19 genes had strong synthetic lethality with Bortezomib (such genes were hereafter designated as synthetic lethal with Bortezomib; SLB). Of the 19 SLB genes, 13 are conserved from yeast to human and include factors involved in ubiquitin/proteasome dependent proteolysis, chromatin silencing, nuclear/cytoplasmic transportation, amino acid and vitamin metabolism, vesicular trafficking, RNA metabolism, etc.

## Results

### Mitotic arrest and failure in chromosome segregation induced by Bortezomib

We found that Bortezomib (LC Laboratories) effectively inhibited proliferation of *S. pombe*, while MG132, an authentic proteasome inhibitor in mammalian cells did not inhibit proliferation (Figure S1). We then examined the level of poly-ubiquitinated proteins in the presence or the absence of Bortezomib (Figure S1). Log-phase cultures were harvested at 0, 4, and 9 hours after addition of 1 mM Bortezomib and total proteins were extracted for immunoblot analysis. In the presence of Bortezomib, poly-ubiquitinated proteins accumulated in a time-dependent manner. Therefore, we concluded that Bortezomib effectively inhibits cellular proliferation and proteolytic activity of the proteasome in *S. pombe*.

**Figure 1:**
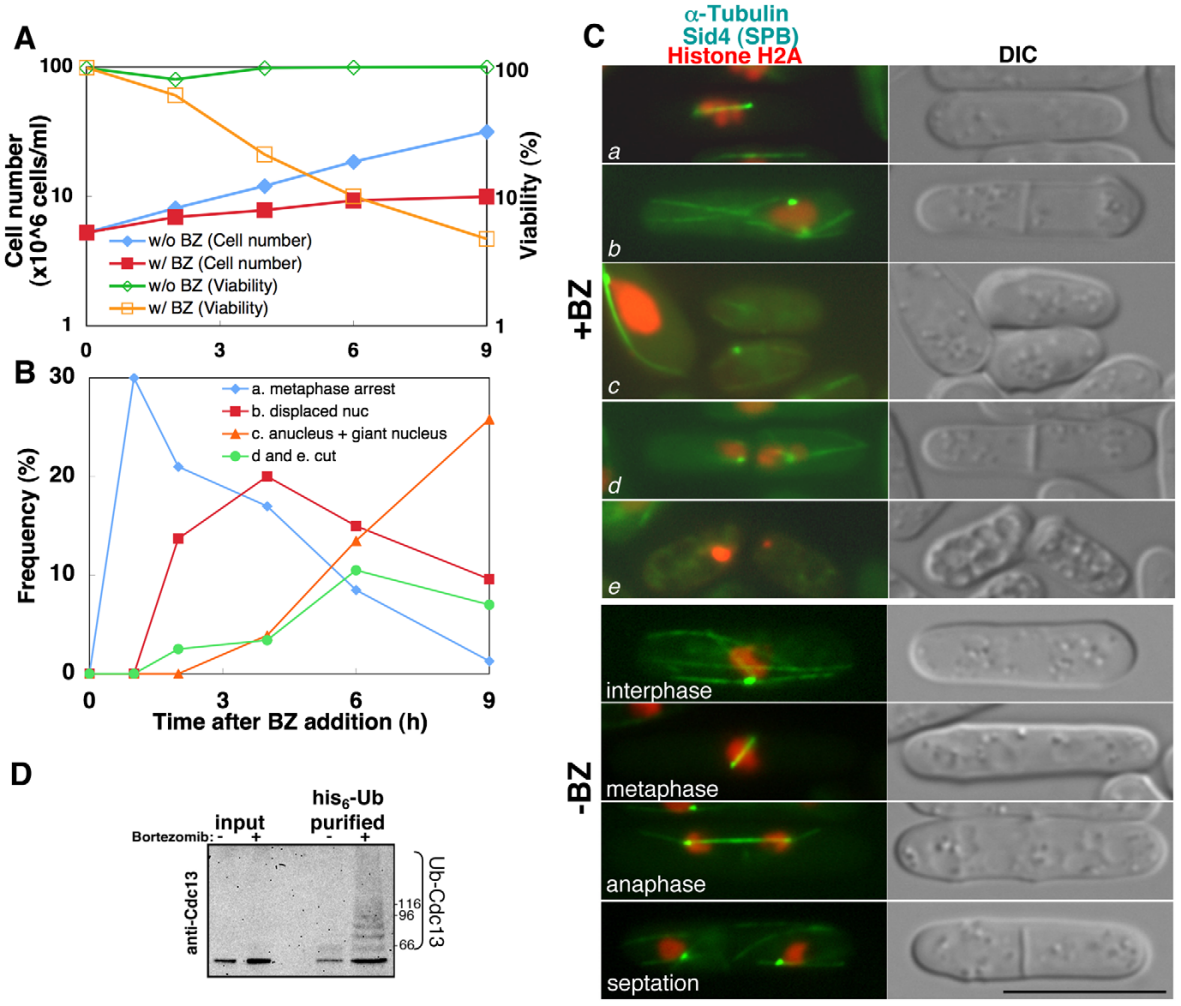
Bortezomib induces metaphase arrest. (**A**) Bortezomib (1 mM) inhibited cellular proliferation. Concentrations of cells and viabilities are presented. BZ: Bortezomib (**B**) Bortezomib (1 mM) inhibited the normal progression of the M phase. The graph indicates the ratio of cells with metaphase spindles and over-condensed chromosomes (blue, cells shown in Figure 1C-*a*), cells with a displaced nucleus (red, Figure 1C-*b*), cells without a nucleus and with a giant nucleus (orange, Figure 1C-*c*), and cells with chromosome torn by the septum (green, Figure 1C-*d*, and *e*). (**C**) Chromosomes, microtubules, and SPB were observed in the presence of Bortezomib. Cells showing mitotic abnormalities correspondent to Figure 2B are shown in a–e (upper panel: +BZ). Images of normal progression of cell division are shown in lower panel (−BZ). Bar = 10 µm (**D**) Poly-ubiquitinated cyclin/Cdc13 accumulated in the presence of Bortezomib. See text for details.

Temperature-sensitive mutants of proteasome components (*mts3-1* for Rpn12 of 19S regulatory particle and *mts2-1* for Rpt2 of 19S regulatory particle) are arrested at M phase due to the inhibition of degradation of mitotic regulators like Cdc13 (cyclin) [10], [11]. This finding led us to examine in detail how Bortezomib affects the cell cycle. After adding Bortezomib to mid log-phase cultures, the cell concentrations and viability were measured over time (Figure 1A). Cell proliferation eventually ceased and viability decreased to 21, 10, and 4.7% at 4, 6, and 9 hours after adding Bortezomib. Without Bortezomib, the cells continued dividing and sustained viability. To examine how the cell cycle was affected by Bortezomib, chromatin DNA, microtubules, and spindle pole bodies (SPB: homologous to centrosome) were visualized by green or red fluorescent protein tagging to histone H2A (for chromatin), alpha-tubulin (microtuble) and Sid4 protein (SPB, the yeast equivalent centrosome, shown as a dot in Figure 1C) respectively. The ratio of cells with over-condensed chromosomes and metaphase spindles was highest (30%) at 1 hour after Bortezomib addition and subsequently decreased (Figure 1B and 1C-a). As the ratio of metaphase cells decreased, the ratio of cells with a displaced nucleus increased (Figure 1C-b), in which sister chromatids were not separated and the nucleus was displaced from the center. As the ratio of ‘displaced nuclei’ was highest at 4 h and decreased subsequently, anucleated cells and cells with a giant nucleus increased (Figure 1B and C-c). This was likely the result of cytokinesis completion in cells with a displaced nucleus. Thus, in the presence of Bortezomib, cells were briefly arrested at metaphase, unable to separate sister-chromatids, and viability was lost. These phenotypes induced by Bortezomib are virtually identical to mitotic defects caused by *mts2-1*, the temperature-sensitive mutation in Rpt2 subunit of 19S particle [10]. In the case of *mts3-1* mutation (Rpn12 of 19S), metaphase arrest phenotype is severer; 75% of cells are briefly arrested at metaphase [11]. In both cases, metaphase arrest is temporal and the nucleus is displaced subsequently as shown in the case of Bortezomib treatment. Given that the defect in the metaphase/anaphase transition is due to the inhibition of proteolysis, ubiquitinated substrates of the proteasome such as Cdc13 (cyclin) should accumulate [12]. To examine this, cells that ectopically expressed hexa-histidine (his6)-tagged ubiquitin were prepared and cultured in the presence or absence of Bortezomib for 4 h at 26°C. The proteins were then extracted from both cultures under denaturing conditions with 6M guanidine-HCl and the resulting extracts were applied to TALON beads (Clontech), which absorb the his6 tag, to purify the ubiquitinated-proteins. TALON-purified proteins were analyzed by immunoblot using an antibody against Cdc13 (Figure 1D). In the presence of Bortezomib, multi-ubiquitinated Cdc13 was observed, as reported in temperature-sensitive mutants of the proteasome [12]. These results demonstrated that a chemical inhibitor for the proteasome can be used to replace the ts proteasome mutants. This drug might be useful to analyze the phenotypes of proteasome-defect at a low temperature, for example, in meiosis or in the experiments in which heat-shock responses should be avoided. Therefore, we adopted Bortezomib for further genetic screening to identify the genes that affect the proteasomal dysfunction phenotype.

### Proteasome-related mutants are hypersensitive to Bortezomib

**Figure 2:**
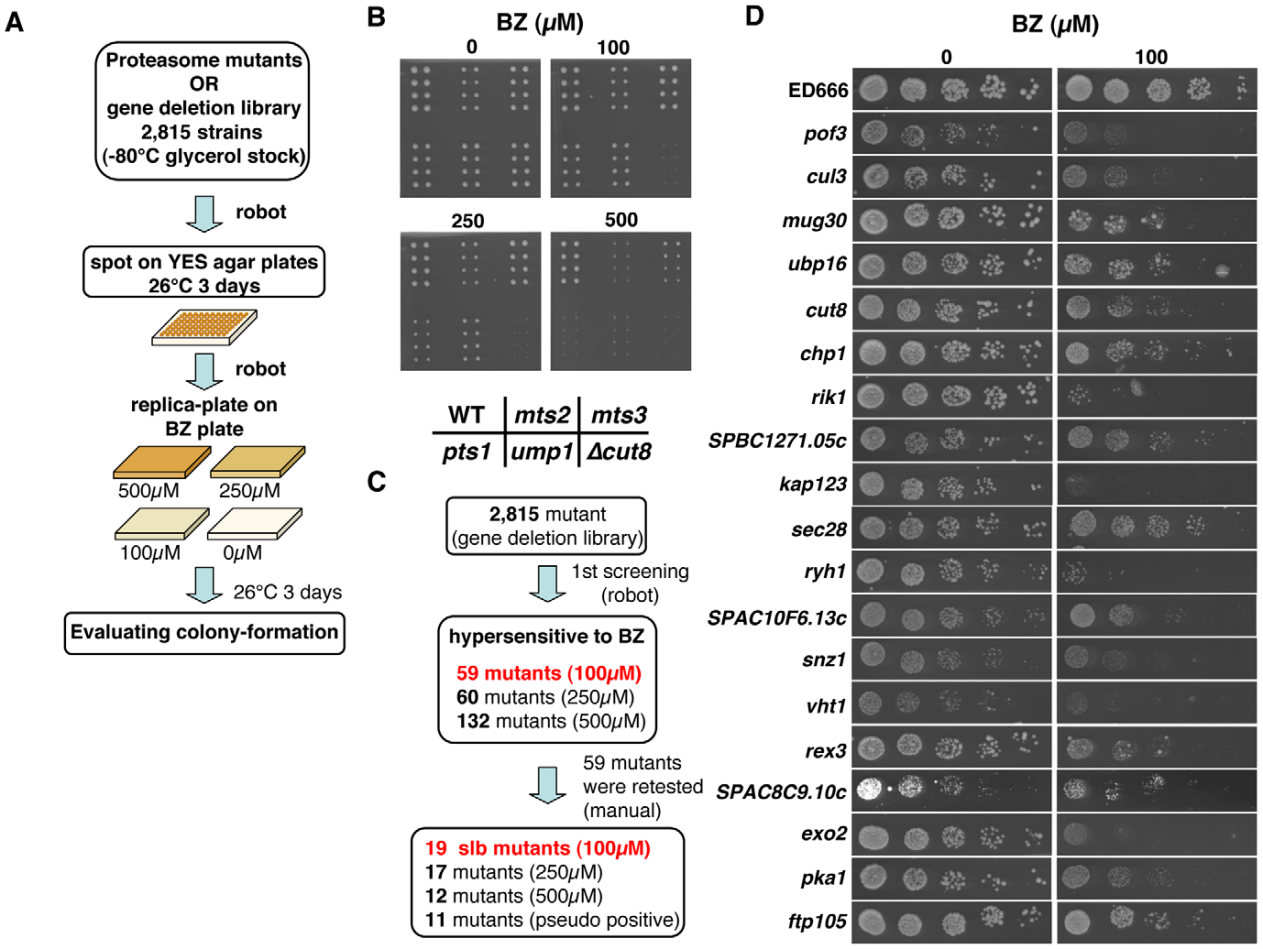
Synthetic lethal screening with Bortezomib. (**A**) Strategy for synthetic lethal screening (**B**) Mutants of components of the ubiquitin/proteasome pathway are hypersensitive to Bortezomib. Eight colonies of each strain were replica-plated onto agar plates with various concentrations of Bortezomib and were incubated for 3 days at 26°C. (**C**) Summary of synthetic lethal screening with Bortezomib. See text for details. (**D**) Validation of isolated mutants that had growth defects in 100 µM Bortezomib by spotting 5-fold serial dilutions of vegetative growing cells.

Prior to the comprehensive screening, we examined how the Bortezomib cytotoxicity is affected by mutations related to the ubiquitin/proteasome system. Compared to the wild type were five proteasome related mutants as follows: *mts2-1*, *mts3-1*, *pts1-727* (mutated in the beta 5 subunit of the 20S complex [13]), *ump1-620* (mutated in the 20S maturation factor Ump1 [13]), and *Δcut8* (gene-deletion mutant of *cut8^+^* required for the proper nuclear localization of the proteasome [14], [15]). Each strain was incubated on a rich YES agar plate to form a colony and then spotted onto agar plates containing 0, 100, 250, or 500 µM Bortezomib, assisted by a robot system (RoToR, Singer, UK). After 3 days incubation at 26°C, the colony formation ability of each stain was evaluated (Figure 2A and B). The wild type formed colonies on all the plates, whereas proteasome-related mutants were defective in colony-formation on Bortezomib plates (*Δcut8* at 100 µM and others at 500 µM). The clear hypersensitivity of *Δcut8* to Bortezomib led us to adopt the above-described method for further genome-wide screening of synthetic lethal mutants with Bortezomib.

### Genome-wide synthetic-lethal screening with Bortezomib

To identify the genes affecting the cytotoxicity of Bortezomib in *S. pombe*, we screened 2815 gene-deletion mutants for synthetic growth inhibition on YES agar plates with Bortezomib using Robot-assisted replica plating. From the primary screening, 59, 62, and 135 strains were isolated that had growth defects in media with 100, 250, and 500 µM Bortezomib, respectively. There was no clear-cut Bortezomib-resistant mutant that grew faster than the wild-type strain on 500 µM Bortezomib medium. A summary of the screening is shown in Figure 2C (examples of raw results of the primary screening and the list of genes are shown in Figure S2 and Table S1). The 59 strains that had growth defects with 100 µM Bortezomib were retested by spotting 5-fold serial dilutions of log-phase cultures of each strain (from 10 cells to 6250 cells) onto YES plates with and without Bortezomib (Figure 2D). We performed these retests in duplicate. As a result, 19 gene-deletion mutants reproducibly showed growth defects on YES plates with 100 µM Bortezomib. Seventeen and 12 strains showed growth defects with 250 and 500 µM Bortezomib, respectively. The rest of the mutants did not show clear growth defects with Bortezomib in the spotting test. None of mutants tested on lower doses (1 nM–10 µM) of Bortezomib showed significant growth defects (Figure S3). Therefore, we adopted a 100 µM concentration of Bortezomib for hypersensitivity screening of *S.pombe* mutants in our present study, although in the previous and similar study on human cells, a 4 to 7-nM concentration of Bortezomib was used for screening [6].

The 19 genes that showed clear growth defects on 100 µM Bortezomib plates in the spotting test were categorized according to function: five belonged to ubiquitin/proteasome pathway, four to nuclear/chromatin proteins and nuclear transport, three to vesicular traffic, three to amino acid and vitamins metabolism, three to RNA metabolism, and protein kinase A (Table 1). Table 1 lists the systematic names, primary names (if applicable), budding yeast *Saccharomyces cerevisiae* and human orthologs, and a short description of each SLB gene. Among the 19 SLB genes, 13 genes are reported to have potential orthologs in humans.

**Table 1:** List of SLB genes.

	*S.pombe*	*S.cerevisiae*	*H.sapiens*	Function
ubiquitin/proteasome	pof3	*DIA2*	STIP1	F-box protein SCF ubiquitin ligase
	cul3/pcu3	*CUL3*	Cullin-3	cullin-RING based BC3B ubiquitn ligase
	mug30	*HUL3*	HECTD2	HECT type ubiquitin ligase
	ubp16	*UBP10*	BAB14306.1	ubiquitin C-terminal hydrolase
	cut8	*STS1/DBF8*		tethering factor for nuclear proteasome
chromatin/nucleus	chp1			chromodomain protein, heterochromatin
	rik1			CLRK ubiquitin ligase complex, gene silencing
	SPBC1271.05c	*YOR052C*		zf-AN1 type zinc finger protein
	kap123	*KAP123/YBR4*	importin-4	Importin beta family
vesicle transport	sec28	*SEC28/ANU1*	coatmer epsilon	vesicle transport
	ryh1/hos1	*YPT6*	Rab-6B	vesicle transport, GTPase, TORC2 regulator
	ftp105	*ECM30*	DMC1	C17orf28/DMC1 ortholog, Golgi localization
metabolism	SPAC10F6.13c	*ASP5/AAT1*	NP002070	pyridoxal phosphate-dependent aminotransferase
	snz1	*SNZ1*		Pyridoxine biosynthesis protein
	vht1	*VHT1*	$	biotin uptake
RNA metabolism	rex3	*REX3*	GOR	Exonuclease, involved in processing of snRNA and rRNA
	SPAC8C9.10c	*RRP14*	SURF6	ribosome biogenesis
	exo2	*KEM1/DST2*	XP033181	Exonuclease II
signal transduction	pka1/git6	*TPK1/2/3*	PKA	cAMP-dependent protein kinase catalytic subunit

$: Five potential orthologs (accession numbers; NP_115671, XP_166184, AA29863, NP_001458.1 and NP_061837) are reported in GeneDB *S. pombe* (Sanger Institute).

## Discussion

In the present study, we demonstrated that Bortezomib, an inhibitor of the proteasome widely used as an anti-cancer drug, effectively inhibits the proliferation of *S.pombe* and induces mitotic arrest as well as temperature-sensitive mutations of the proteasomal subunits. Nineteen gene deletion mutants were identified by the genome-wide screening to be synthetic lethal with Bortezomib.

Despite the strong effect of Bortezomib to arrest the cell cycle, another proteasome inhibitor, MG132, had weaker inhibitory effects on proliferation in the present study. MG132 is, however, reported to inhibit protesome-dependent proteolysis in the cell lysate of *S.pombe*, indicating that the proteasome of *S.pombe* is sensitive to this inhibitor [16]. In *S.cerevisiae*, MG132 is used to inhibit proteolysis *in vivo* under the gene deletion of PDR5, the major drug efflux pump, which might effectively excrete MG132 from the cell [17]. *S.pombe* possesses two PDR5 orthologs, Pdr1 and Bfr1. The difference in the effects of Bortezomib and MG132 might be due to their differences in cell permeability or the efficacy of excretion by drug efflux pumps. Bortezomib may serve as a useful tool to study the ubiquitin/proteasome pathway in *S.pombe*.

**Figure 3:**
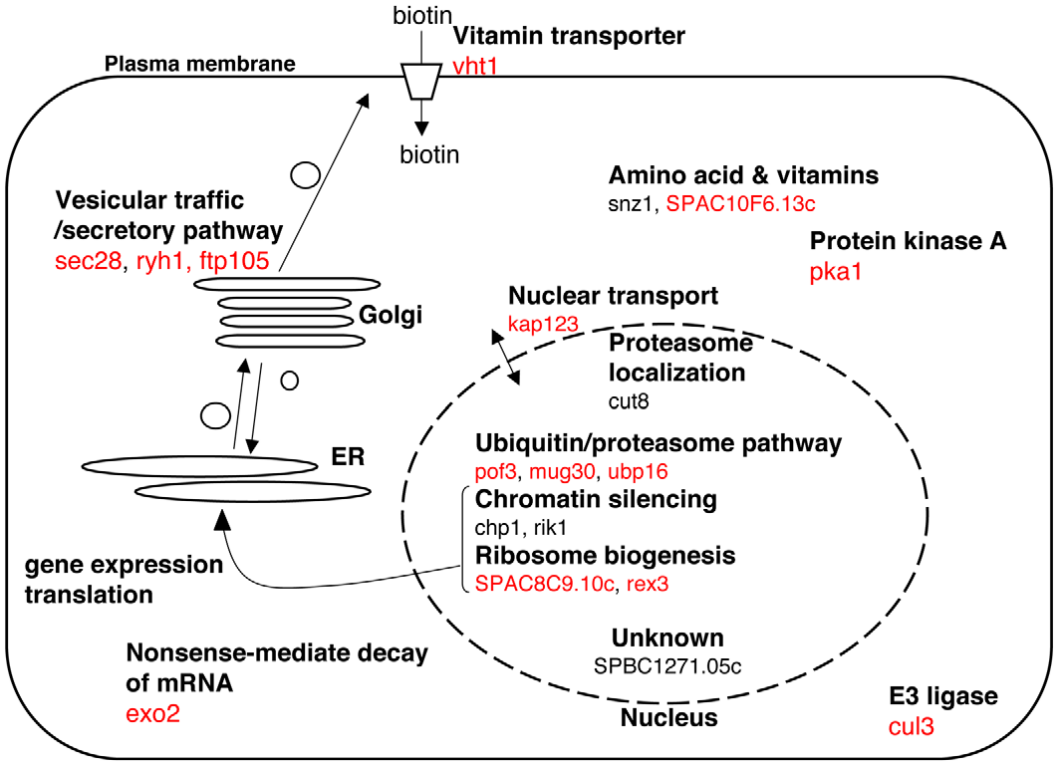
Summary of identified SLB genes. Thirteen conserved SLB genes are shown in red.

We performed the synthetic-lethal screen to identify genes that affect sensitivity for Bortezomib, using the 2815 gene-deletion mutants of *S. pombe*. Nineteen deletion mutants were identified with severe growth defects induced by 100 µM Bortezomib (listed in Table 1 and Figure 3). Five of the responsible genes (designated SLB) were ubiquitin/proteasome-related: pof3, cul3, mug30, ubp16 and cut8. Their synthetic lethality with Bortezomib might be explained through the drug's inhibitory action against proteasome. For example, ubiquitin ligases provide the substrates for proteasome so that diminishing both might cause severe synthetic effects. Others have not been reported to be related to proteasome function. However, some of SLB genes could still be explained through the proteasome functions. For three vesicular trafficking SLB genes (sec28, ftp105, and ryh1), defects in secretory pathway invoke ER (endoplasmic reticulum) stress that may enhance requirement of the proteasome activity [18]. One of vesicular trafficking SLB genes, ftp105, encodes Golgi localizing protein that was reported to interact with deubiquitinase Usp5 and be required for the Golgi localization of Usp5 [19]. The human ortholog of Ftp105 is C17orf28/DMC1 (down-regulated in multiple cancers), a potential tumor suppressor [20]. Therefore, the synthetic lethality of ftp105 deletion with Bortezomib will be studied further in future. Ryh1 was recently reported to regulate TORC 2 (target of rapamycin complex 2) in *S.pombe* [21]. Concerning nuclear SLB proteins, more proteasome might be required when the chromatin dynamics is compromised in deletion mutants of chromatin regulators, as the nuclear proteasome is known to contribute to chromatin regulations like DNA damage repair, DNA replication, and transcription [15], [22], [23], [24]. One of nuclear SLB gene products is Rik1, a component of CLRK ubiquitin ligase complex required for chromatin silencing [25], [26]. Although substrates of CLRK ubiquitin ligase are not known, it may be a curious experiment to examine whether Bortezomib affects DNA chromatin silencing. PKA was reported to be involved in metaphase/anaphase regulations in the fission yeast and in Xenopus-egg systems *in vitro* [12], [27], [28]. While some of the other SLB genes, such as vitamin metabolic factors, are difficult to be explained, they might be implicated to one of very diverse cellular functions of the proteasome. Bortezomib may possibly have targets other than the proteasome within cells of *S. pombe*. Thus evaluation of SLB genes apparently unrelated to ubiquitin/proteasome might be worth for considering other targets. Combination of SLB gene deletion and proteasomal temperature-sensitive mutations will be useful to judge whether the synthetic lethality is due to inhibition of the proteasome or to other perturbations caused by Bortezomib. If the synthetic lethality is due to inhibition of the proteasome, the double mutant of the SLB gene and the proteasome is expected to show a much severer phenotype than a single proteasomal mutant. Actually, a mutant of *cut8*, an SLB gene, shows synthetic lethality to proteasomal mutations *mts2-1* and *mts3-1* [14]. Although it should be kept in mind that Bortezomib has another target, the present results have potentially important implications for basic proteasome biology by opening avenues for discovering novel and unexpected relationships between the proteasome and other cellular pathways. A similar genome-wide screen in human cells suggested that protein translations, ER/Golgi pathway, DNA damage repair pathway, and regulation of Myc and polyamines are involved in Bortezomib-induced cell death [6]. The findings of the present study newly suggest that genes involved in vitamin and amino acid metabolic pathways, chromatin silencing, nuclear/cytoplasm shuttling, and the cAMP pathway are related to the proteasome in the fission yeast *S.pombe*. Therefore, further efforts must be made to understand the mechanisms of the synthetic lethality of these unexpected SLB gene deletions with Bortezomib.

We identified 13 conserved SLB genes potentially interesting for further studies. As mentioned in the Results, 4 to 7 nM of Bortezomib was used to screen genes affecting the cytotoxicity of Bortezomib in cancer cell lines, and 100 µM of Bortezomib was used for *S.pombe* mutant screening in the present study. The difference in Bortezomib sensitivity might reflect the difference in the biology of these organisms, such as drug permeability and drug excretion. In general, yeast cells are more resistant to perturbations by chemical inhibitors. Therefore, human orthologs of conserved SLB genes should be examined by small interference-RNA to see whether their knockdown affects the survivability of human cells at lower doses of Bortezomib. If the same synthetic effects occur in human cells, such SLB genes have potential for the innovation of new therapies or diagnoses. For example, if chemical inhibitors for these conserved SLB products are developed, such chemicals will be candidates used for cocktail therapy with Bortezomib. On the other hand, patients with a genetically weak background due to these SLB orthologs might have severe adverse effects upon Bortezomib administration. Thus further investigations on the SLB orthologs in human are expected in future.

## Materials and Methods

### Strain, medium, culture, and drug treatments

*S. pombe* heterothallic haploids 972*h^−^* and 975*h^+^* and their derivatives were used. Complete rich YE, YES and minimal EMM2 media were used [29]. Stock solutions of Bortezomib (LC Laboratories, Woburn, MA) were prepared in DMSO and drugs were added to liquid culture or agar medium at the indicated concentration.

### Synthetic lethal screening

For genome-wide screening, we adopted the deletion library of the *S. pombe* haploid purchased from Bioneer Corp. (Korea). The control wild type strains are ED666 (*h^+^ ade6-M210 ura4-D18 leu1-32*) and ED668 (*h^+^ ade6-M216 ura4-D18 leu1-32*), which were also purchased from Bioneer Corp. The haploid gene-deletion library was provided as glycerol stocks in 96-well plates. First, 5 µl of each stock of deletion strain was spotted onto YES plates from a 96-well plate using the laboratory automation system BioMek FX (Beckman Coulter, Brea, CA). After 3 days incubation at 26°C, a colony of each strain was picked-up and spotted onto another YES plate (considered the mother plates) using the RoToR robot (Singer Instruments, UK). One colony was quadruplicated to check reproducibility. From the mother plates, spotted colonies were again picked-up and spotted onto YES plates containing 0, 100, 250, and 500 µM Bortezomib, respectively. Spotted plates with various concentrations of the drug were incubated for 3 days at 26°C and the colony formation of each strain was evaluated. For validation of the primary screening, Bortezomib sensitivities of selected strains from the primary screening were retested using spotting tests.

### Immunoblot and protein purification

For immunoblot analysis, total proteins were extracted using the trichloroacetic acid (TCA) method. Identical amounts of proteins were separated by SDS-PAGE gel and blotted to nitrocellulose membranes. Anti-poly-Ubiquitin (FK-2; mouse monoclonal, MBL, Japan), anti-alpha-tubulin (TAT1; mouse monoclonal, a gift from Dr. Gull) and anti-Cdc13 (rabbit polyclonal) were used as primary antibodies. Horseradish peroxidase-conjugated secondary antibodies and an ECL chemiluminescence system (GE Healthcare) were used to amplify signal expression. To purify ubiquitinated proteins, the previously described method was applied with minor modification [15].

### Fluorescent microscopy

All images were acquired using a fluorescent microscope setting AxioPlan 2 (Zeiss, Germany). Methods of construction of GFP or RFP fused gene were previously described [30].

## Supporting Information

Figure S1 **Bortezomib inhibits proliferation of** ***S.pombe*** **.** (A) Bortezomib and MG-132 were added to a log-phase culture of *S. pombe* at the indicated concentrations and cellular proliferation was examined for 8 hours. Fold-increases at 8 hours after drug addition are presented on the Y-axis. (B) Levels of poly-ubiquitinated proteins were examined in the presence (+) or absence (−) of 1 mM Bortezomib. Poly-ubiquitinated proteins accumulated in a time-dependent manner after the addition of Bortezomib.
(TIF)

Figure S2 **An example of the primary screening is shown.** As described in the text, every colony of each gene-deletion strain was spotted to each position (A1, A2…) of YES agar plates with 0, 100, 250, and 500 µM Bortezomib. To screen 2815 strains, 31 sets of these plates were prepared. After incubating at 26°C for 3 days, colony formation was evaluated. Wild-type strains were spotted to positions H2 and H3 (white broken line). Strains spotted onto A3, B8, B10, and C10 were selected as candidates showing severe growth defects with 100 µM Bortezomib and were retested by serial dilution spotting.
(TIF)

Figure S3 **Sensitivity to lower doses of Bortezomib.** Colony-formation ability of five slb mutants was examined on YES agar medium containing 0, 1 nM, 10 nM, 100 nM, 1 µM, 10 µM, and 100 µM Bortezomib as described in Figure 2 (D). Under 10 µM Bortezomib, significant growth defect was not observed.
(TIF)

Table S1 **List of genes that were identified to show growth defect in the presence of 100, 250 and 500 µM Bortezomib from the primary screening.**
(XLS)
