# Supplementary figures and images for: Identification of Genes Affecting the Toxicity of Anti-Cancer Drug Bortezomib by Genome-Wide Screening in S. pombe
Source: PLoS One. 2011 Jul 8;6(7):e22021. doi: 10.1371/journal.pone.0022021 (PMC3132776; doi:10.1371/journal.pone.0022021)

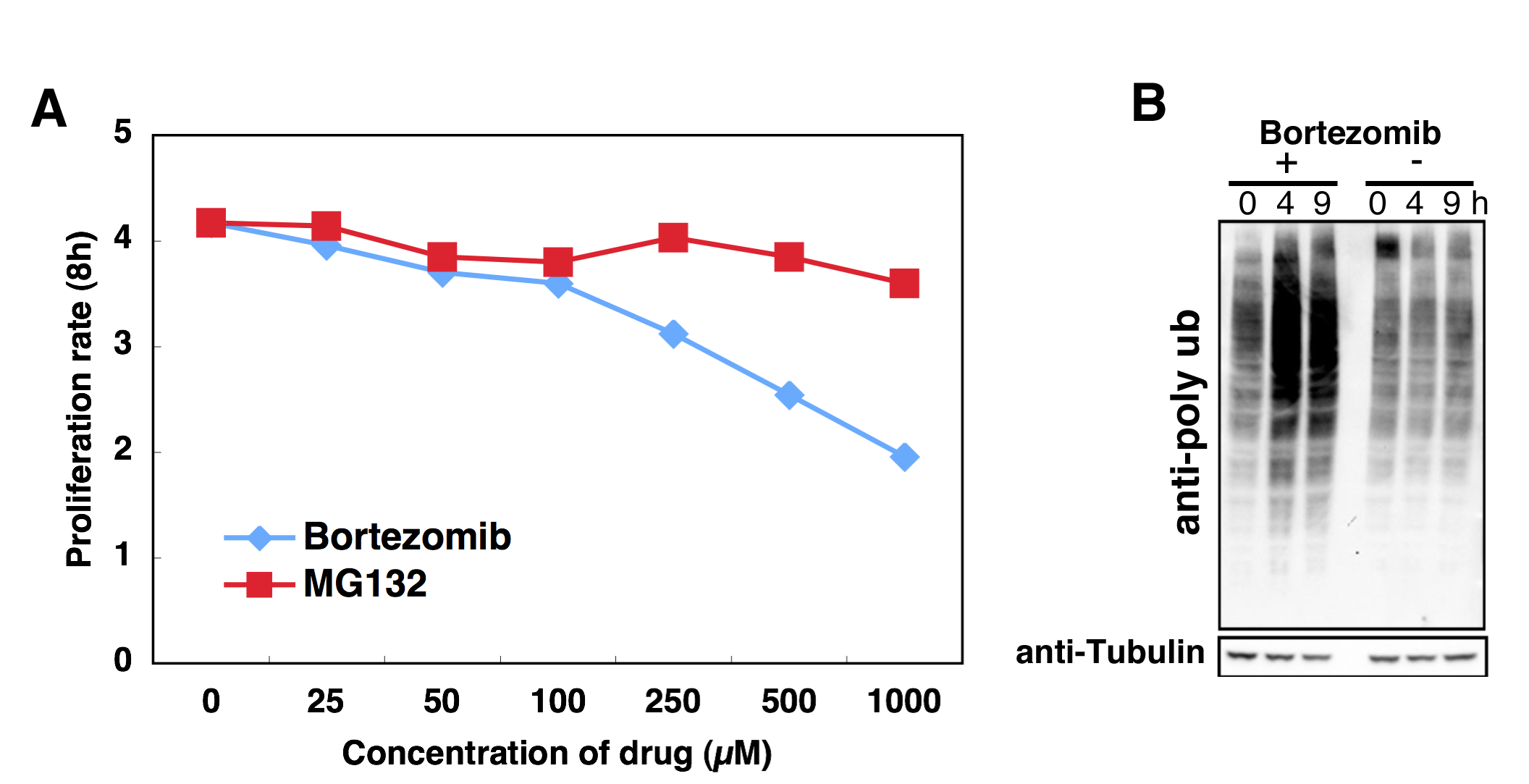

Supplement: Figure S1 — Bortezomib inhibits proliferation of S.pombe . (A) Bortezomib and MG-132 were added to a log-phase culture of S. pombe at the indicated concentrations and cellular proliferation was examined for 8 hours. Fold-increases at 8 hours after drug addition are presented on the Y-axis. (B) Levels of poly-ubiquitinated proteins were examined in the presence (+) or absence (−) of 1 mM Bortezomib. Poly-ubiquitinated proteins accumulated in a time-dependent manner after the addition of Bortezomib. (TIF) [file pone.0022021.s001.tif]

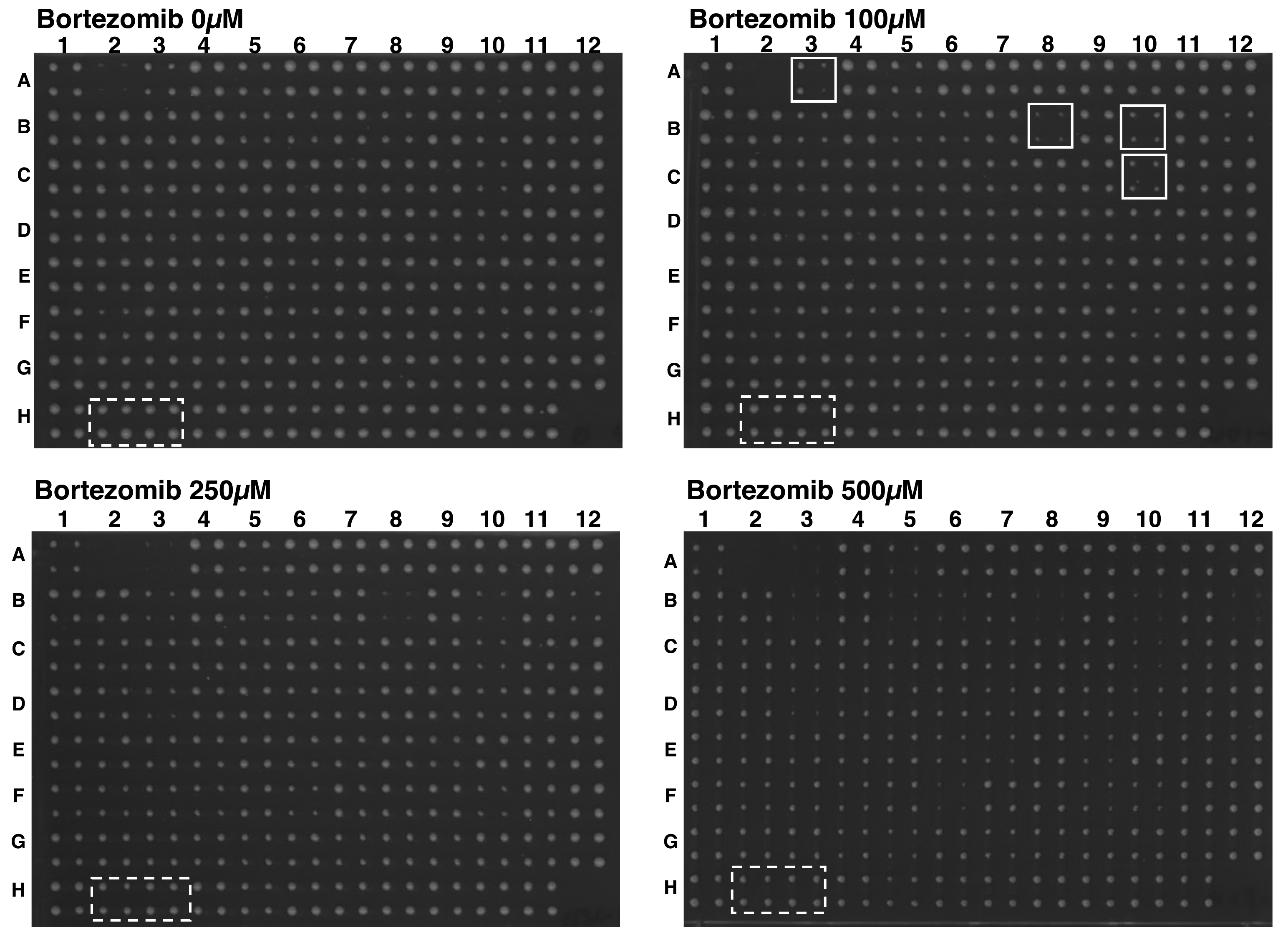

Supplement: Figure S2 — An example of the primary screening is shown. As described in the text, every colony of each gene-deletion strain was spotted to each position (A1, A2…) of YES agar plates with 0, 100, 250, and 500 µM Bortezomib. To screen 2815 strains, 31 sets of these plates were prepared. After incubating at 26°C for 3 days, colony formation was evaluated. Wild-type strains were spotted to positions H2 and H3 (white broken line). Strains spotted onto A3, B8, B10, and C10 were selected as candidates showing severe growth defects with 100 µM Bortezomib and were retested by serial dilution spotting. (TIF) [file pone.0022021.s002.tif]

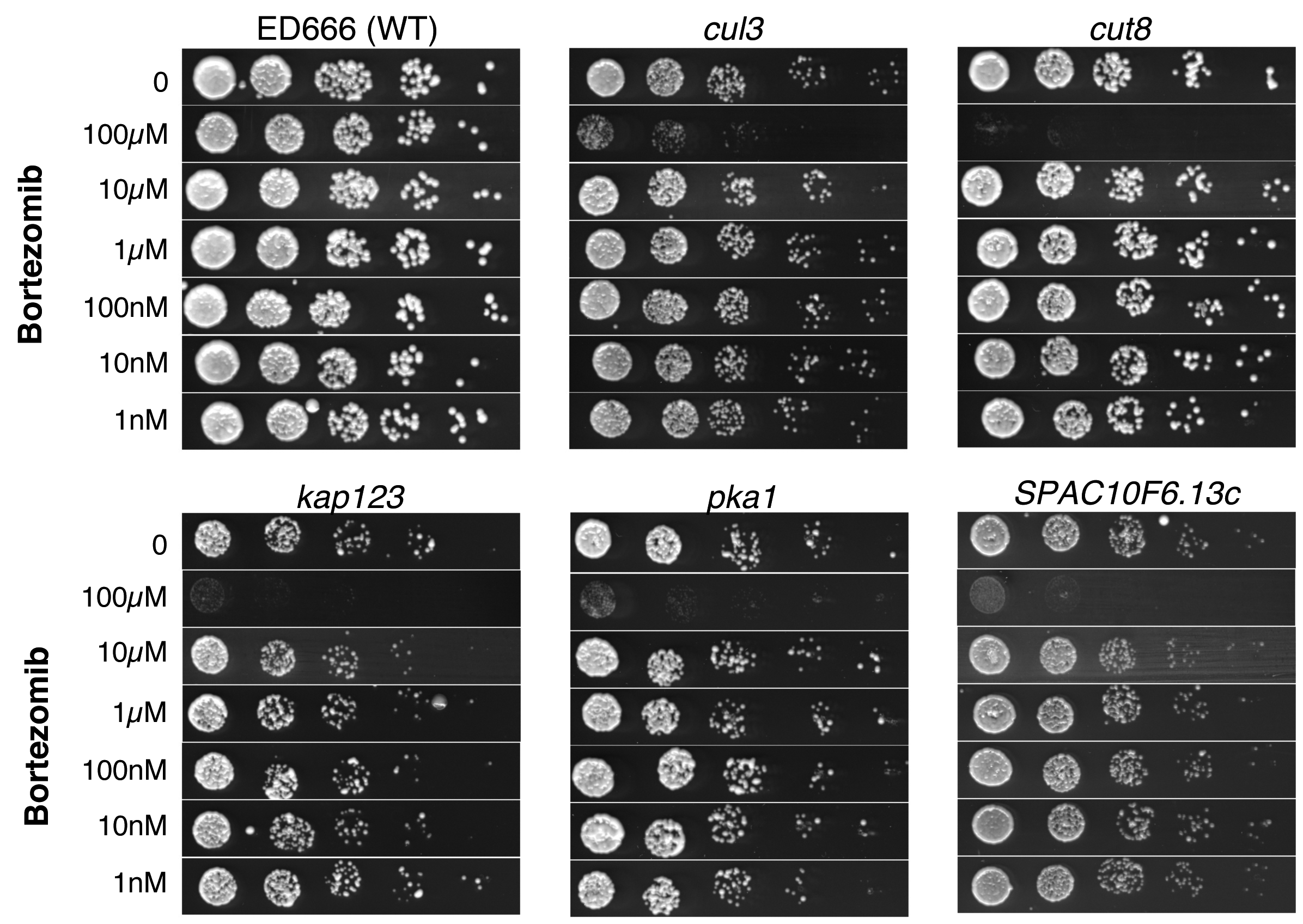

Supplement: Figure S3 — Sensitivity to lower doses of Bortezomib. Colony-formation ability of five slb mutants was examined on YES agar medium containing 0, 1 nM, 10 nM, 100 nM, 1 µM, 10 µM, and 100 µM Bortezomib as described in Figure 2 (D). Under 10 µM Bortezomib, significant growth defect was not observed. (TIF) [file pone.0022021.s003.tif]
